# An integrative ultrastructural and transcriptomic analysis of host–pathogen interactions with human brain microvascular endothelial cells during cryptococcal infection: a preliminary study

**DOI:** 10.3389/fcimb.2026.1735570

**Published:** 2026-02-09

**Authors:** Priya Krishnan, Gautham Arunachal Udupi, Rashmi Santhoshkumar, Manjunatha. M. V., Veena Kumari H. B., Netravathi, Nagarathna S.

**Affiliations:** 1 Dept of Neuromicrobiology, National Institute of Mental Health and Neuro Sciences, Institute of National Importance, Bengaluru, India; 2 Dept of Human Genetics, National Institute of Mental Health and Neuro Sciences, Institute of National Importance, Bengaluru, India; 3 Dept of Neuropathology, National Institute of Mental Health and Neuro Sciences, Institute of National Importance, Bengaluru, India; 4 Dept of Neurovirology, National Institute of Mental Health and Neuro Sciences, Institute of National Importance, Bengaluru, India; 5 Dept of Neurology, National Institute of Mental Health and Neuro Sciences, Institute of National Importance, Bengaluru, India

**Keywords:** blood-brain barrier, cryptococcal meningitis, differential gene expression, dual RNAseq, HBMECs, host-pathogen interaction, invasion, transmission electron microscopy

## Abstract

**Background:**

In humans, *Cryptococcus neoformans* and *Cryptococcus gattii* species complexes are the leading cause of fungal meningitis globally. To establish CNS infection, *Cryptococcus* must breach the BBB, primarily comprised of specialized brain microvascular endothelial cells (BMECs), which is the prerequisite for cryptococci to invade the brain. Despite its clinical impact, the mechanisms underlying host–pathogen interaction at the BBB particularly involving environmental isolates remain under-characterized.

**Objective:**

This study aimed to investigate the cyto-morphological and transcriptomic responses of HBMECs to infection by clinical and environmental *Cryptococcus* isolates using a dual approach—ultrastructural electron microscopy and high-throughput dual RNA-Seq.

**Methods:**

HBMECs were infected *in vitro* with molecularly typed clinical and environmental isolates of *C. neoformans* and *C. gattii* at two infection time points (4 hpi and 18 hpi). Transmission electron microscopy was used to visualize host cell ultrastructural alterations, while dual RNA-Seq was performed to assess differential gene expression in both host and pathogen.

**Results:**

TEM revealed extensive ultrastructural changes in infected HBMECs, including membrane ruffling, increased microvilli, mitochondrial alterations, ER dilation, Golgi fragmentation, nuclear deformation, and autophagosome formation. Transcriptomic profiling demonstrated functional enrichment of several critical cryptococcal virulence-associated genes linked to immune evasion and stress adaptation including various immune signaling pathways elicited by the HBMECs as a counter measure to the cryptococcal invasion.

**Conclusion:**

Clinical and environmental *Cryptococcus* isolates exhibit comparable invasive potential and elicit similar host endothelial responses with consistent effects observed across all isolates and time points. This integrative study combining ultrastructural and transcriptomic analyses highlights conserved host-pathogen interactions at the BBB, identifies potential molecular targets for antifungal therapy and underscores the pathogenic relevance of environmental reservoirs in cryptococcal meningitis. cryptococcal meningitis, blood-brain barrier, invasion, transmission electron microscopy, Dual RNASeq, differential gene expression, host-pathogen interaction, HBMECs, ultrastructural alterations.

## 1 Introduction

Cryptococcosis, the disease caused by the environmental fungi *Cryptococcus neoformans* and *Cryptococcus gattii*, typically manifests as severe pulmonary infection and can progress to cryptococcal meningitis, a serious condition affecting the central nervous system. These pathogens primarily affect individuals with compromised immune systems, but an increasing number of cases have also been reported in immunocompetent hosts contributing significantly to global morbidity and mortality (Maziarz and Perfect, 2015; Diniz-Lima et al., 2022). The infection is primarily acquired by the inhalation of desiccated airborne yeast cells which establishes itself within the alveoli and may remain dormant for years and later on spread to other organs more often the CNS owing to its neurotropism (Negroni and Arechavala, 2015; Taylor-Smith, 2017). Invasion of *Cryptococcus* into the CNS necessitates crossing the Blood–brain barrier (BBB) (Casadevall et al., 2018) which serves as a critical interface between the systemic circulation and the CNS regulating the passage of molecules and pathogens into the brain (Kadry et al., 2020; Zhou et al., 2024).

In their natural habitat, *C.neoformans* species complex (serotypes A and D) are most frequently isolated from soil contaminated with pigeon guano and other avian habitats and has also been recovered from desiccated accumulations of bird droppings. Conversely, *C.gattii* species complex (serotypes B and C) has been associated with dried and decayed materials of *Eucalyptus camaldulensis* and pine trees (Diniz-Lima et al., 2022). In this study, we isolated *C.neoformans* and *C.gattii* from these ecological sources. These environmental isolates lack prior human host exposure, unlike clinical *Cryptococcus* isolates that have encountered the challenging conditions within the human host. A key event in cryptococcal invasion involves the interaction of the fungal pathogen with the brain endothelial cells and adapts itself to survive within the nutrient-deprived environment of the brain. During the cryptococcal invasion into BBB, significantly marked morphological changes was observed in the endothelial cells indicating that *Cryptococcus* induced host cell actin cytoskeletal reorganization (Galán and Zhou, 2000; Chen et al., 2002; Eugène et al., 2002; Chen et al., 2003). May et al., in their comprehensive review, described numerous discoveries that highlight the intricacies of interactions between the host and *Cryptococcus* influencing the disease severity (May et al., 2016). In addition, previous experimental studies have demonstrated unique expression profiles among different mutants or reference cryptococcal strains under various conditions (Steen et al., 2003; Florio et al., 2011; Tavares et al., 2019; Yu et al., 2021). A study conducted by Yu et al. comparing the transcriptomic variations of clinical and environmental isolates of *C.neoformans*, highlighted on genes with lineage-specific expression providing insights into genes important for *in vitro* and *in vivo* growth stages (Yu et al., 2020).

However, while considerable research has explored the behavior of laboratory or clinical isolates of *Cryptococcus in vitro* and *in vivo*, relatively little is known about the virulence potential of environmental isolates, particularly in terms of their ability to invade brain endothelial cells and the resulting host response. In this context, our study investigates whether environmental isolates of *C.neoformans* and *C. gattii* possess invasive capabilities comparable to clinical isolates in breaching the BBB endothelium and characterize the associated ultrastructural and transcriptional responses of HBMECs during fungal invasion. To achieve this, we employed an integrative approach combining high-resolution transmission electron microscopy (TEM) with dual RNA sequencing (RNA-Seq).

This study represents a preliminary yet comprehensive effort to provide foundational insights into early endothelial responses to infection by clinical and environmental *Cryptococcus* isolates, enhancing our understanding of host–pathogen interactions at the BBB using an *in vitro* co-culture system. TEM was employed to examine host cellular architecture, revealing changes in membrane dynamics, organelle morphology, and structural adaptations induced by fungal invasion. Complementing this, high-throughput dual RNA sequencing enabled simultaneous profiling of host and pathogen gene expression, uncovering differential expression of virulence-associated fungal genes and activation of diverse immune signaling pathways in HBMECs. This integrative approach provides critical insights into the cellular and molecular mechanisms underlying cryptococcal invasion of the BBB.

## 2 Materials and methods

### 2.1 Clinical and environmental isolates of *Cryptococcus* species complex

The study was conducted in the Department of Neuromicrobiology at the National Institute of Mental Health and Neurosciences (NIMHANS), Bengaluru. Only laboratory-confirmed cases of cryptococcal meningitis established by cryptococcal antigen detection and fungal culture were included. The experimental work was conducted using *Cryptococcus* isolates recovered from the diagnostic CSF samples obtained from patients of all age groups. The isolates were further confirmed using MALDI -TOF MS or VITEK 2 compact system. The study was conducted in compliance with approvals obtained from the Institutional Ethics Committee and Institutional Biosafety Committee.

The environmental isolates of *Cryptococcus* were recovered from dried pigeon droppings and decayed material from Eucalyptus trees (flower, bark, leaves and soil contaminated with bird guano) as per the protocol outlined in the previous studies (Pham et al., 2014; Nweze et al., 2015) subsequently identified by standard conventional and automated methods.

### 2.2 *In vitro* monolayer cell culture of HBMECs

HBMECs were a generous and kind contribution by late Dr. Kwang Sik Kim, Professor of Pediatrics, Johns Hopkins University, Baltimore, USA and Dr. Anirban Banerjee, Professor, IIT, Mumbai towards the proposed objectives. The anchorage dependent HBMECs were seeded onto treated cell culture petri plates supplemented with growth media RPMI 1640 (Gibco) containing 10% FBS (Gibco), 10% Nuserum (Corning), 1% Minimum Essential media (Gibco), 0.5% Pen-Strep (Gibco) and incubated at 37^0^ C with 5%CO_2_ maintained (Jong et al., 2008). The plates were periodically monitored to ensure healthy growth and confluence with particular attention towards depletion of nutritional resources.

### 2.3 Infection of HBMECs with *Cryptococcus*

The isolates were selected (**Table 1**) for infecting HBMECs based on their molecular types, determined using the PCR fingerprinting technique with a synthetic oligonucleotide, (GTG)5 primer, following the protocol outlined by Tay et al (Tay et al., 2006). The molecular type of each isolate was assigned by comparison with the control strains of *Cryptococcus* corresponding to VNI-VNIV, VNB and VGI-VGIV. As the HBMEC monolayers reached approximately 70–80% confluence, cells were infected with *Cryptococcus neoformans/gattii* isolates derived from both clinical and environmental sources. Yeast cells were grown to the early logarithmic phase in YEPD broth (HiMedia), harvested, washed, and resuspended in endothelial growth medium. The fungal inoculum was quantified using a hemocytometer and adjusted to achieve a final concentration of approximately 1 × 10^6^ CFU/mL corresponding to a multiplicity of infection (MOI) of ~10 as described previously (Lahiri et al., 2019). Infected cultures were incubated at 37°C in a humidified atmosphere containing 5% CO_2_.

**Table 1:** List of clinical and environmental isolates of *Cryptococcus* selected based on their molecular types to infect HBMECs.

Sl No.	Isolates	Isolate ID	Isolate Name	Molecular type
1.	Clinical isolates	CL_Cneo_35	*C. neoformans*	VN II
2.		CL_Cneo_52	*C. neoformans*	VN I
3.		CL_Cgattii_76	*C. gattii*	VG IV
4.		CL_Cgattii_77	*C. gattii*	VG IV
5.	Environmental isolates	EN_Cneo_12	*C. neoformans*	VN I
6.		EN_Cneo_24	*C. neoformans*	VN II
7.		EN_Cgattii_26	*C. gattii*	VG IV
8.		EN_Cgattii_28	*C. gattii*	VG IV

The study included a total of eight *Cryptococcus* isolates, comprising two isolates each of *C.neoformans* and *C. gattii* from clinical sources and two isolates each of *C. neoformans* and *C. gattii* from environmental sources (n = 8; **Table 1**). At the end of 4^th^ and 18^th^ hour of incubation the cells were gently washed with PBS to remove unbound and extracellular yeast. Following incubation cells were harvested for downstream analyses. Invasion was confirmed by CFU enumeration on Sabouraud dextrose agar following Triton X-100 lysis. Infected cultures were analyzed and compared to uninfected controls at two distinct time points to assess morphological alterations and differential gene expression in both the host and pathogen.

### 2.4 Cyto-morphological study by transmission electron microscopy

Transmission electron microscopy was performed in duplicate independent experiments on HBMECs infected with *Cryptococcus neoformans/gattii* isolates derived from both clinical and environmental sources (n = 8 isolates; **Table 1**) at 4 and 18 hpi, along with uninfected controls. The HBMECs at the end of 4^th^ and 18^th^ hpi and the uninfected controls were primarily fixed in 4% paraformaldehyde and 2.5% glutaraldehyde. The cells were secondarily fixed in 1% Osmium tetroxide and dehydrated with graded series of distilled ethanol (70%, 80%, 90%, 95% & 100%) for 1hour each at 4^0^C. Clearing of the cells in propylene oxide was performed twice. Infiltration with propylene oxide: Araldite resin (1:1 ratio) for overnight followed by increasing proportion of araldite (1:2 & 1:3 ratios) for one hour respectively. Finally, the cell pellets were embedded in pure resin and polymerized at 60^0^C.

Using ultramicrotome (Leica UC7, Germany) ultrathin sections were collected on copper grids and stained using Leica EM AC20 automatic contrasting system (Leica, Germany). Saturated methanolic uranyl acetate (TAAB, UK) and 3% aqueous lead citrate (Ultrostain 2) were used for staining. For each experimental condition, 4–5 ultrathin-section grids were prepared and multiple microscopic fields were examined at varying magnifications to capture representative ultrastructural features. Imaging was performed using the transmission electron microscope JEM 1400-Plus, JEOL (Tokyo, Japan).

### 2.5 Dual RNA seq and bioinformatics

#### 2.5.1 RNA extraction and sequencing

RNA was extracted from 16 co-cultures and 5 baseline controls using the Qiagen RNeasy Mini Kit. RNA quality and concentration were assessed using Nanodrop spectrophotometry (*A*_260_/*A*_280_ purity ratio of ~2.0), agarose gel electrophoresis, and Agilent Bioanalyzer to determine RNA integrity (RIN score). Libraries were prepared from high-quality RNA using the TruSeq Stranded mRNA kit (Illumina), with fluorometric quantification using Invitrogen Qubit Fluorometer. A total of 42 cDNA libraries (16 co-cultures and 5 baseline controls in duplicate) were constructed and the library quality was validated using the Bioanalyzer DNA 1000 chip. Libraries were normalized, pooled, denatured, and sequenced in replicates on the Illumina NextSeq 550 platform using high-throughput massively parallel sequencing technology.

#### 2.5.2 Bioinformatics pipelines

The initial raw sequences obtained from the sequencing facility were presented as FastQ files in a single end configuration with 76 base pair reads. A subset of FASTQ files were submitted to the BLAST database to check for the possible contamination and confirm that the hits were in agreement with the expected organism. The quality of the reads was assessed using FASTQC and Cutadapt to remove the poor quality reads and adapter/ primer sequences. The dual RNASeq data were further filtered based on the read length and quality scores. The high quality reads with Phred score >30 were considered and mapped to the human reference genome and the unmapped reads were aligned to the second reference genome of *Cryptococcus* species in the RNA-Seq Alignment workflow using STAR aligner. *Cryptococcus neoformans / gattii* species complex reference genome was generated using the RNA custom genome builder app version 2.0.2 by obtaining the FASTA and GTF genome annotated file formats from Fungi Ensembl database. The aligned read counts were then quantified using RNA differential expression workflow that enables the detection of differentially expressed genes by employing DESeq2 pipeline. The statistical package R was used for visualizing the gene expression patterns and to identify the differentially expressed genes in both the pathogen (yeast) and the host (HBMECs) and the results were obtained as graphs and tables. Further the web based tool DAVID was used for the functional enrichment analysis of DEGs extracted by Gene Ontology and the significantly altered pathways associated with the DEGs were determined using KEGG pathway maps for the host. We performed the functional categorization of differentially expressed genes of fungal pathogen by using Gene Ontology employing FungiDB and FungiFun 2.2.8 BETA web based tools (Priebe et al., 2015).

#### 2.5.3 qPCR validation

A total of nine genes, including both HBMECs (FOS, FOSB, DDIT4, VEGFA, TRIB3, FOS, FOSB) and *Cryptococcus* (CGB_A4480W, CGB_A4770W) transcripts, were selected for validation using quantitative real-time PCR (qPCR). Total RNA used for sequencing and qPCR was DNase-treated and reverse transcribed to cDNA using the Takara cDNA conversion kit following the manufacturer’s instructions. qPCR reactions were performed in duplicate with 20 ng/μL cDNA in a 25 μL reaction using Takara SYBR Green master mix (Agilent Technologies, USA) on an Agilent AriaMx Real-Time PCR system. The cycling conditions included initial denaturation at 95°C for 30 sec, followed by 40 cycles of 95°C for 5 sec and 60°C for 34 sec. The dissociation curve analysis confirmed primer specificity. Relative expression levels were calculated using the 2^-ΔΔCt method. β-actin for HBMECs and CGB_Act for *Cryptococcus* served as reference genes for normalizing the qPCR experiment. Pearson’s correlation test was used to assess concordance between RNA-Seq and qPCR data.

#### 2.5.4 Comparison categories

To mark the expression differences among various entities the interacting co-cultures were compared to the baseline controls at two different time points. Therefore, the following groupings were assigned for the clearer perception of the comparisons made (**Table 2**).

**Table 2:** Challenged HBMECs co-culture categories.

Treated Co-culture categories	Description
CLCneo_T4	HBMECs infected with clinical isolate of *C.neoformans* retrieved at 4^th^ hpi
CLCneo_T18	HBMECs infected with clinical isolate of *C.neoformans* retrieved at 18^th^ hpi
CLCgattii_T4	HBMECs infected with clinical isolate of *C.gattii* retrieved at 4^th^ hpi
CLCgattii_T18	HBMECs infected with clinical isolate of *C.gattii* retrieved at 18^th^ hpi
ENCneo_T4	HBMECs infected with environmental isolate of *C.neoformans* retrieved at 4^th^ hpi
ENCneo_T18	HBMECs infected with environmental isolate of *C.neoformans* retrieved at 18^th^ hpi
ENCgattii_T4	HBMECs infected with environmental isolate of *C.gattii* retrieved at 4^th^ hpi
ENCgattii_T18	HBMECs infected with environmental isolate of *C.gattii* retrieved at 18^th^ hpi

## 3 Results

### 3.1 Cyto-morphological changes of challenged HBMECs

**Figure 1:**
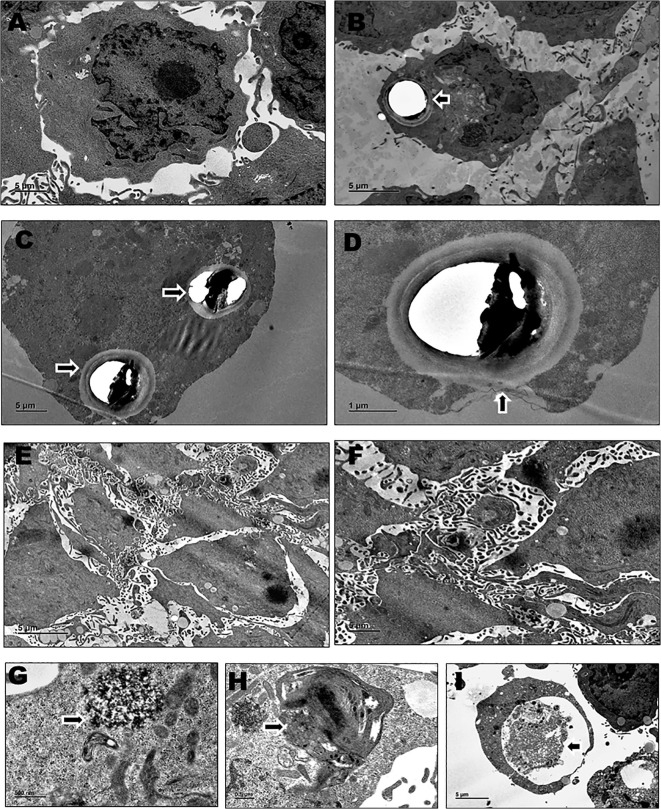
The electron micrographs shown are representative images of HBMECs obtained from duplicate independent experiments performed by infecting with *Cryptococcus neoformans/ gattii* species complex isolates derived from both clinical and environmental sources. For each experimental condition, 4–5 ultrathin-section grids were examined, and multiple microscopic fields (~10–12 fields) were analyzed at the indicated magnifications. **(A)** uninfected HBMEC (x1500); **(B)** endothelial cell with invaded *Cryptococcus* (x1200); **(C)** endothelial cell containing two *Cryptococcus* within (x1500); **(D)** endothelial cell exhibiting membrane invagination during the engulfment of *Cryptococcus* (x4000); **(E, F)** HBMECs showing membrane ruffling and extensive microvilli formation (x800, x1500); **(G– I)** HBMECs undergoing autophagy showing the presence of autophagic vacuoles or autophagosomes (x8000, x5000, x1200).

In their uninfected state, HBMEC displayed typical elongated endothelial morphology with relatively smooth, flat cell membrane and a spherical to oval shaped nucleus. Intracellular organelles such as mitochondria, Golgi complex, ER and nucleus were identifiable. Upon exposure to *Cryptococcus*, there were substantial degenerative changes observed in the regular morphology of HBMECs. The electron micrographs, **Figures 1B, C** depicts the presence of *Cryptococcus* yeast cells that have invaded the microvascular endothelial cells. **Figure 1D** offers a view of the active involvement of the endothelial cell membrane in engulfing the yeast cell exhibiting striking membrane invagination and the extension of microvilli projections to encapsulate the invading *Cryptococcus*.

The sub microscopic preparations of infected HBMECs revealed notable membrane ruffling and extensive formation of microvilli (**Figures 1E, F**). In contrast, the uninfected HBMECs showed the minimal presence of microvilli (**Figure 1A**) as these structures are part of their physiological characteristics that aid in increasing the cell surface area. Interestingly, we noticed the presence of irregular and large protrusions, folds or undulations on the cell membrane of infected HBMECs referred to as membrane ruffles. Remarkably, we observed HBMECs exhibiting autophagy in response to cryptococcal infection as an important cellular defense mechanism. Membrane bound vesicles known as autophagic vacuoles or autophagosomes were formed within the endothelial cell that probably involves the degradation and recycling of cellular components, such as damaged/ vacuolated organelles and intracellular pathogens (**Figures 1G, H, I**).

### 3.2 Ultrafine subcellular structural alterations

#### 3.2.1 Mitochondria

**Figure 2:**
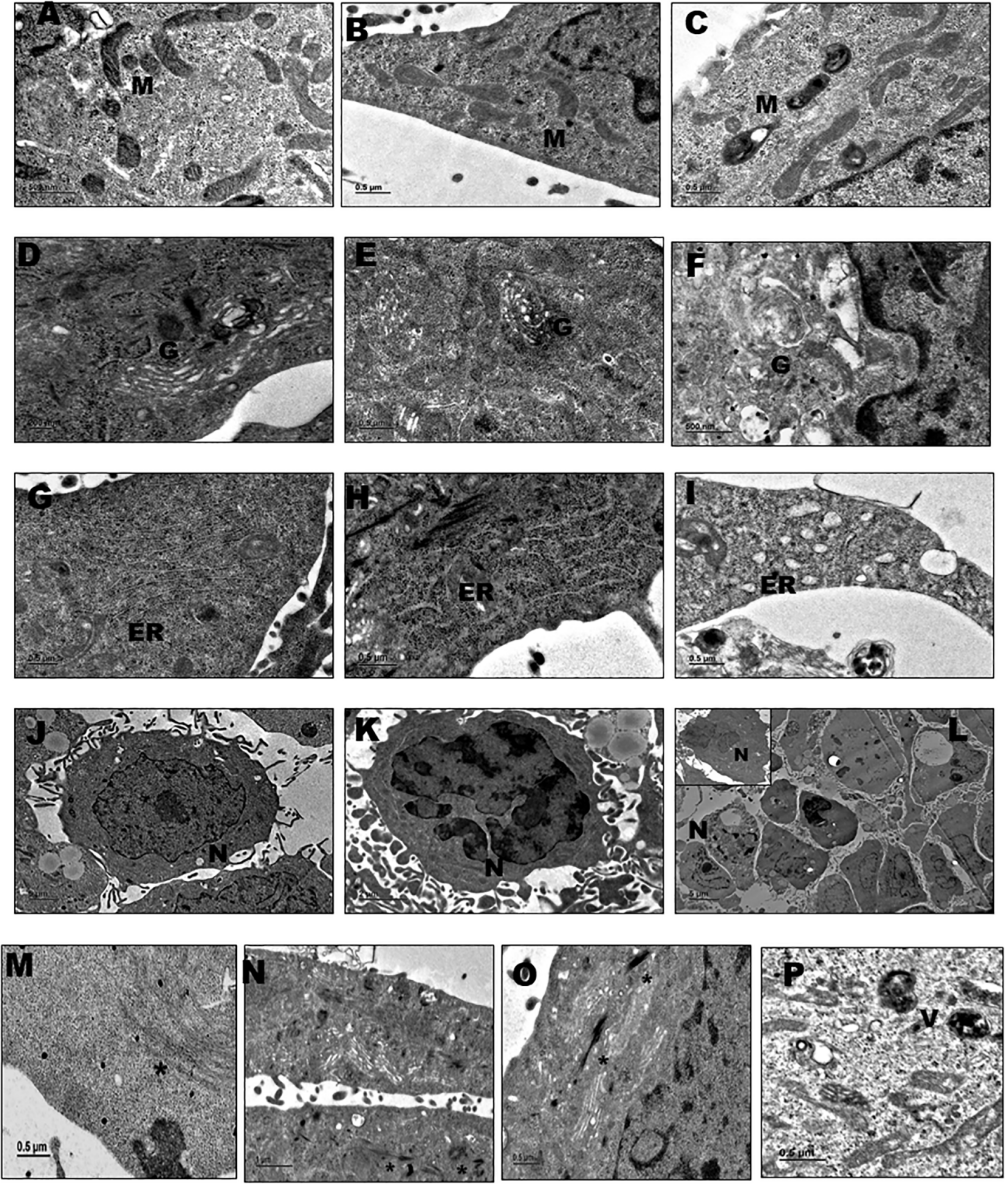
The electron micrographs shown are representative images of HBMECs obtained from duplicate independent experiments performed by infecting with *Cryptococcus neoformans/gattii* species complex isolates derived from both clinical and environmental sources showing subcellular changes at 4th and 18th hour post infection. For each experimental condition, 4–5 ultrathin-section grids were examined and multiple microscopic fields (~10–12 fields) were analyzed at the indicated magnifications. **(A)** Mitochondria **(M)** in uninfected HBMECs (x8000); **(B)** alterations in mitochondria at 4th hpi (x6000); **(C)** alterations in mitochondria at 18th hpi (x6000); **(D)** Golgi apparatus **(G)** in uninfected HBMECs (x10000); **(E)** alterations in Golgi at 4th hpi (x6000); **(F)** alterations in Golgi at 18th hpi (x8000); **(G)** Endoplasmic reticulum (ER) in uninfected HBMECs (x5000); **(H)** alterations in ER at 4th hpi (x6000); **(I)** alterations in ER at 18th hpi (x6000); **(J)** Nuclear morphology **(N)** in uninfected HBMECs (x1500); **(K)** alterations in nuclear morphology at 4th hpi (x2000); **(L)** alterations in nuclear morphology at 18th hpi (x500, x1200); **(M)** actin filaments (*) in uninfected HBMECs (x5000); **(N)** stress fibers (*) at 4th hpi (x4000); **(O)** stress fibers (*) at 18th hpi (x6000). **(P)** Vacuoles (V) within the host cell cytoplasm (x6000).

In their uninfected state, HBMECs exhibited typical mitochondrial morphology characterized by intact membranes and a well-defined cristae pattern (**Figure 2A**). However, upon infection with *Cryptococcus*, there was a profound and degenerative impact on the mitochondrial morphology. At 4^th^ hour time point post infection, mitochondria displayed partial alterations with slight dilated appearance and less distinguishable cristae pattern (**Figure 2B**). In contrast, at 18^th^ hour infection time point, there was significant loss of mitochondrial integrity accompanied by aberrant architectural remodeling, appearing invariably swollen with distorted morphology, the cristae pattern was completely disrupted, membranes seemed irregular and detached. Furthermore, the mitochondria appeared vacuolated and there was decrease in matrix density, all indicative of severe mitochondrial dysfunction (**Figure 2C**).

#### 3.2.2 Golgi apparatus

In their non-infected state, HBMECs displayed the characteristic Golgi structure with flattened, membrane bound cisterns stacked in series along with budding vesicles (**Figure 2D**). However, at the 4th-hour time point after infection with *Cryptococcus*, there was a discernible but relatively minor alteration in the regular Golgi morphology (**Figure 2E**). A more significant change occurred by the end of the 18-hour infection period, characterized by marked dilation and fragmentation of the Golgi cisternae (**Figure 2F**). Portions of the Golgi apparatus appeared to have redistributed from their typical perinuclear location to positions closer to the periphery of the endothelial cell, becoming dispersed throughout the cell cytoplasm.

#### 3.2.3 Endoplasmic reticulum

The Endoplasmic Reticulum (ER) in uninfected HBMECs exhibited regular morphology comprising flattened, interconnected membrane structures characterized with ribosomes on its cytoplasmic surface, which imparts a rough appearance when observed under an electron microscope (**Figure 2G**). However, following *Cryptococcus* infection, significant changes in the ER morphology became apparent. At the 4th-hour time point post-infection, the ER retained a structure resembling its typical appearance (**Figure 2H**). Nonetheless, by the end of 18-hour infection period, the ER underwent substantial fragmentation and dilation, accompanied by the formation of ER-derived vesicles. These ER fragments were found dispersed throughout the cellular cytoplasm. Notably, it was observed that the ribosomes present on the surface of the rough endoplasmic reticulum with dilated lumen, had become partially detached (**Figure 2I**) and appeared more similar to the smooth endoplasmic reticulum in its morphology. These structural alterations in the ER have the potential to significantly impact the cellular processes related to protein synthesis, folding, and post-translational modifications affecting the overall cellular homeostasis.

#### 3.2.4 Nucleus

The nuclear morphology of HBMECs typically entails round to oval shape with a smooth and regular contour, along with a prominent nucleolus located within the nucleoplasm (**Figure 2J**). However, cryptococcal infection induced significant irregularities in the nuclear morphology of HBMECs. By the end of the 4th hpi, the nuclear shape exhibited alterations with the initiation of fragmentation, presenting multiple indentations (**Figure 2K**). By the 18th hpi, nuclear fragmentation had advanced, resulting in the presence of 2–3 distinct fragments, each displaying an irregular morphology. Notably, the nuclear membrane was poorly defined and had suffered complete disruption (**Figure 2L**). Overall, the endothelial cells appeared to be undergoing early stages of apoptosis, reflecting the cellular response to the cryptococcal infection.

#### 3.2.5 Cytoplasm

Generally, the cytoskeleton fibers are arranged in a parallel orientation along the length of the cell (**Figure 2M**). However, following infection their morphology and arrangement were altered. As the infection progressed, these fibers became denser and formed more compact bundles, with the effect being more pronounced at 18th hour (**Figure 2O**) time point compared to the 4th hpi (**Figure 2N**). The presence of vacuoles or membrane-bound compartments was observed within the host cell cytoplasm which could be formed as a result of altered metabolic processes or cellular stress induced by the infection (**Figure 2P**). Such vacuoles may contain various materials, including organelles, proteins, or pathogen-related components suggesting that the host may be attempting to restrict the pathogen's spread and limit its impact as a defensive mechanism.

### 3.3 Submicroscopic morphological modifications within HBMECs in response to clinical and environmental *Cryptococcus* isolates

The ultrastructural changes observed in HBMECs at the 4^th^ and 18^th^ hour time points following infection with clinical and environmental isolates of *Cryptococcus* yielded consistent and indistinguishable results. These degenerative changes displayed a resemblance in response of HBMECs to isolates from both the source, suggesting a parallel impact of *Cryptococcus* on subcellular morphology during the early and later phases of infection. The infected HBMECs exhibited initial signs of morphological modifications, relatively minor subcellular organelle alterations at 4^th^ hpi. This early response was consistent across both clinical and environmental isolates, suggesting a commonality in the impact of *Cryptococcus* on subcellular morphology during the initial phases of infection. As the infection progressed to the 18th-hour time point, both clinical and environmental isolates induced severe subcellular organelle degenerative changes and dysfunction in HBMECs. These changes were uniform across *Cryptococcus* isolated from both clinical and environmental source, emphasizing the robustness of the host cell's response to the fungal infection. Membrane ruffling, extensive microvilli formation and autophagy were uniformly observed within HBMECs challenged with clinical and environmental *Cryptococcus* isolates, signifying a synchronized cellular response where the host attempts to counteract the infection.

### 3.4 Dual RNASeq of the infected host and the pathogen

Dual RNASeq is a powerful high through put omics approach to gauge the gene expression profiles simultaneously of the infecting pathogen and the host being infected. The quality of RNAs as estimated by Bioanalyzer calculating the RIN ranged from 8.4–10 indicating least degraded and the most quality RNA. All the libraries yielded fragment size considered optimal, ranged from 289–375 nt and the sequencing of each sixteen co-culture specimens in duplicates generated 25 – 38.8 million reads. In total, 38,552; 1,014; and 763 genes were mapped to the human, *C.neoformans* and *C.gattii* reference genomes respectively. The analysis employed cut off of ≤ 0.05 for p-values, ≤ 0.05 for false discovery rate (FDR) and an absolute log2FoldChange value ≥ 1.5 for up regulated and ≤ -1.5 for down regulated significant profiling of the genes that are differentially expressed.

### 3.5 Evaluation of differential gene expression in HBMECs

**Figure 3:**
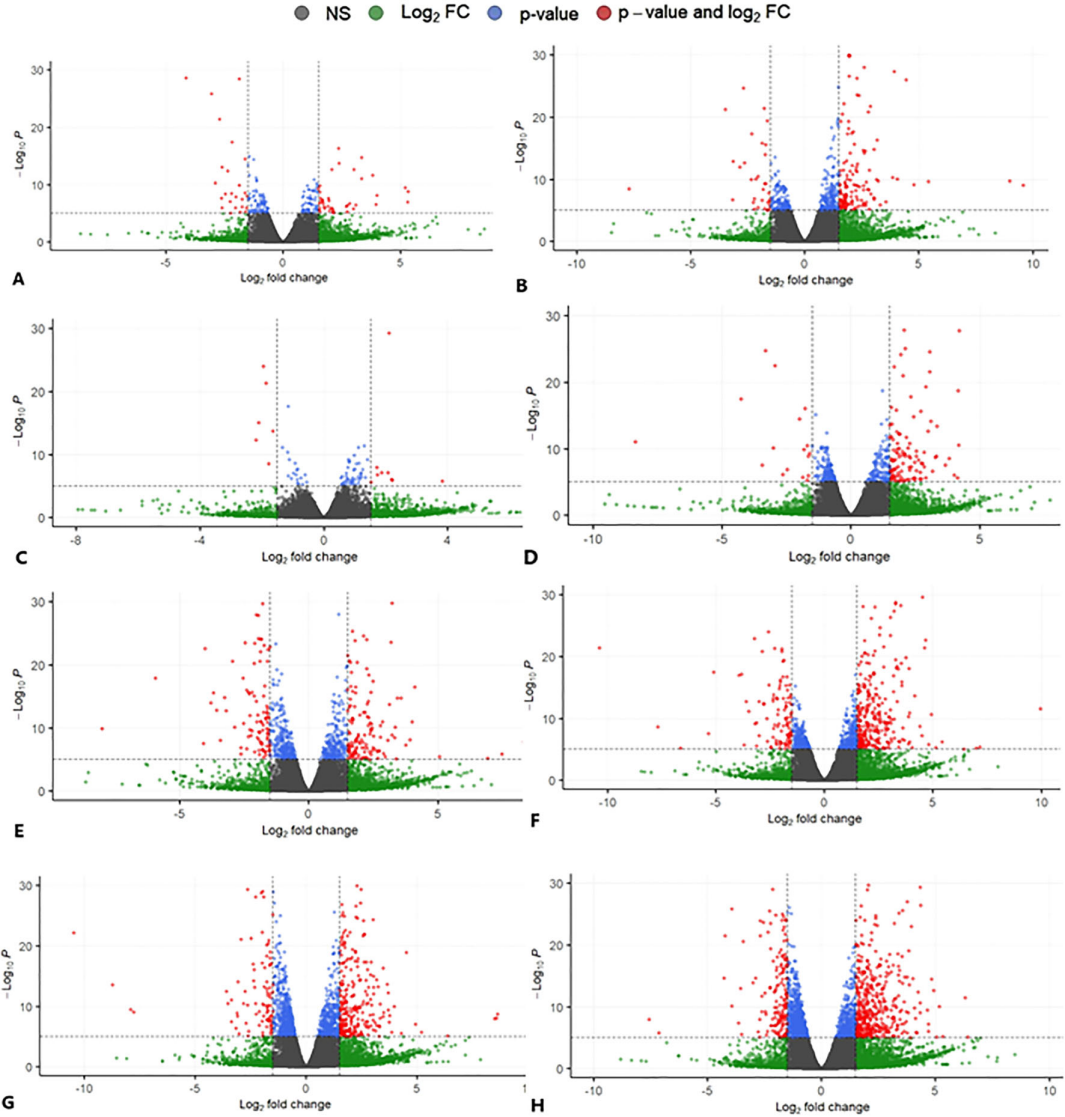
Volcano plot depicting the gene expression profiles of all the eight treated categories **(A–H)**, where the variables passing both the statistical significance criteria with p-value cut off (≤ 0.05) and Log2FC cut off (≥ 1.5, ≤ -1.5) are represented in red. The challenged HBMECs as described under **Table 2** **(A–H)** was compared to the uninfected HBMECs (host) to profile the various differentially expressed genes during the event of host and the pathogen interaction measured at two different time points (T4 and T18) *in vitro*. The data shown are representative of duplicate independent RNA-Seq experiments conducted under identical experimental conditions.

**Figure 4:**
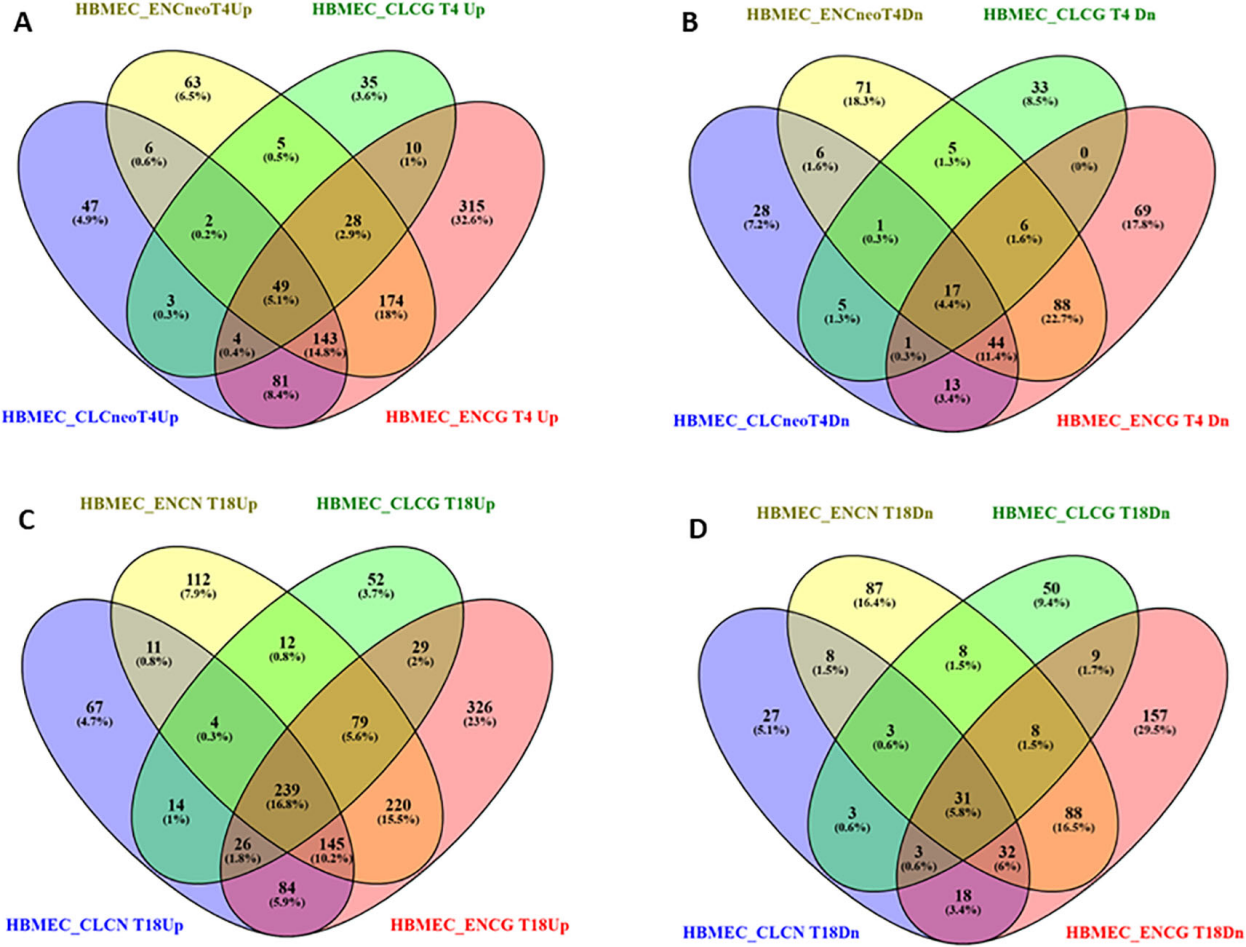
Venn diagram visually displaying the shared and unique sets of differentially expressed up-regulated **(A)** (4th hpi) **(C)** (18th hpi)] and down-regulated **(B)** (4th hpi) **(D)** (18th hpi)] genes in HBMECs (host) obtained by comparing all the eight challenged categories with uninfected HBMECs describing the common and distinct DEGs across different comparison categories. The non-overlapping numbers represent elements unique to each condition while the overlapping ones signify mutually shared DEGs among the various comparison entities. The data shown are representative of duplicate independent RNA-Seq experiments conducted under identical experimental conditions.

To elucidate the gene expression responses exhibited by the host upon encountering the pathogen, sequenced reads from all the described eight co-culture categories were compared with the reads from the uninfected HBMECs. The comparative analysis revealed a range of DEGs encompassing diverse functional categories in the host (**Figure 3**). Of the 3275 (CLCneo_T4), 4876 (CLCneo_T18), 2391 (CLCgattii_T4), 4031 (CLCgattii_T18), 5093 (ENCneo_T4), 5659 (ENCneo_T18), 6292 (ENCgattii_T4), 6790 (ENCgattii_T18) genes found to be differentially expressed in the host among the various treated categories; we found 335, 590, 136, 455, 470, 822, 804, 1148 significantly upregulated genes and 115, 125, 68, 115, 238, 265, 238, 346 significantly downregulated genes sequentially with respect to all the eight treated categories in response to the infecting pathogen on the basis of selection criteria for DEGs (**Figure 4**).

Infection of HBMECs with clinical and environmental isolates of *C.neoformans* and *C.gattii* resulted in 49 (5.1%) and 239 (16.8%) commonly upregulated genes at time points T4 and T18, respectively. Similarly, 17 (4.4%) and 31 (5.8%) genes exhibited common downregulation at T4 and T18 time points. Furthermore, the comparative analysis led to the identification of uniquely up and down regulated elements among all the eight infected co-culture categories: 47 (4.9%), 67 (4.7%), 35 (3.6%), 52 (3.7%), 63 (6.5%), 112 (7.9%), 315 (32.6%), 326 (23%) and 28 (7.2%), 27 (5.1%), 33 (8.5%), 50 (9.4%), 71 (18.3%), 87 (16.4%), 69 (17.8%), 157 (29.5%) respectively in comparison with the non-infected HBMECs (**Figure 4**). The genes displaying differential expression were categorized into three primary functional Gene Ontology groups namely biological processes, cellular components, and molecular functions.

### 3.6 Gene ontology enrichment analysis of DEGs in HBMECs

The functional enrichment analysis of genes differentially expressed at the early infection time point (4 hrs) revealed enrichment in several biological processes and molecular functions with genes coding for proteins involved in integrin-mediated cell adhesion (VTN, ITGB3, ITGB8, EMILIN2, FBN1), immune responses (CCL5, TNFSF13, IFITM2, ITGB8, C3, TRIM22), reactions to toxic substances (ADAMTS13, CCL5, CYP1B1, NUPR1, SLC7A11), regulation of macrophage chemotaxis (CCL5, IL34, TRPV4), apoptotic processes (BACE1, DDIT3, GRIK5, PCSK9, NUPR1, BBC3), autophagy-related functions (DDIT3, TP53INP1, RAB39B, TRIB3, NUPR1), T-cell migration (CCL5, DOCK8, ITGB3, TNFRSF14), control of angiogenesis (PTGIS, HSPB6, ADM2, ITGB3, ITGB8, EMILIN2, CYP1B1, TLR3, RAPGEF3, AQP1, VEGFA), cellular responses to hypoxia (VCAM1, MB, DDIT4, SLC2A1, CYP1A1, MTHFR, CA9, ABAT, CRYAB, LOXL2, VEGFA), and glycolytic processes (ACTN3, DDIT4, NUPR1).

Notably, the cellular response to interleukin 1, IL-2, IL-9, IL-15, and IL-4 exhibited significant upregulation in the host as an initial reaction to the invading fungal pathogen. In Ambrose Jong et al.'s time-course study, a genomic analysis of HBMECs infected with *C.neoformans* showed consistent alterations in MHC gene expression throughout the interactions at all stages, suggesting that host cells undergo significant gene profile changes and play an active role in CNS immunoregulation during *C.neoformans* infection (Jong et al., 2008).

**Figure 5:**
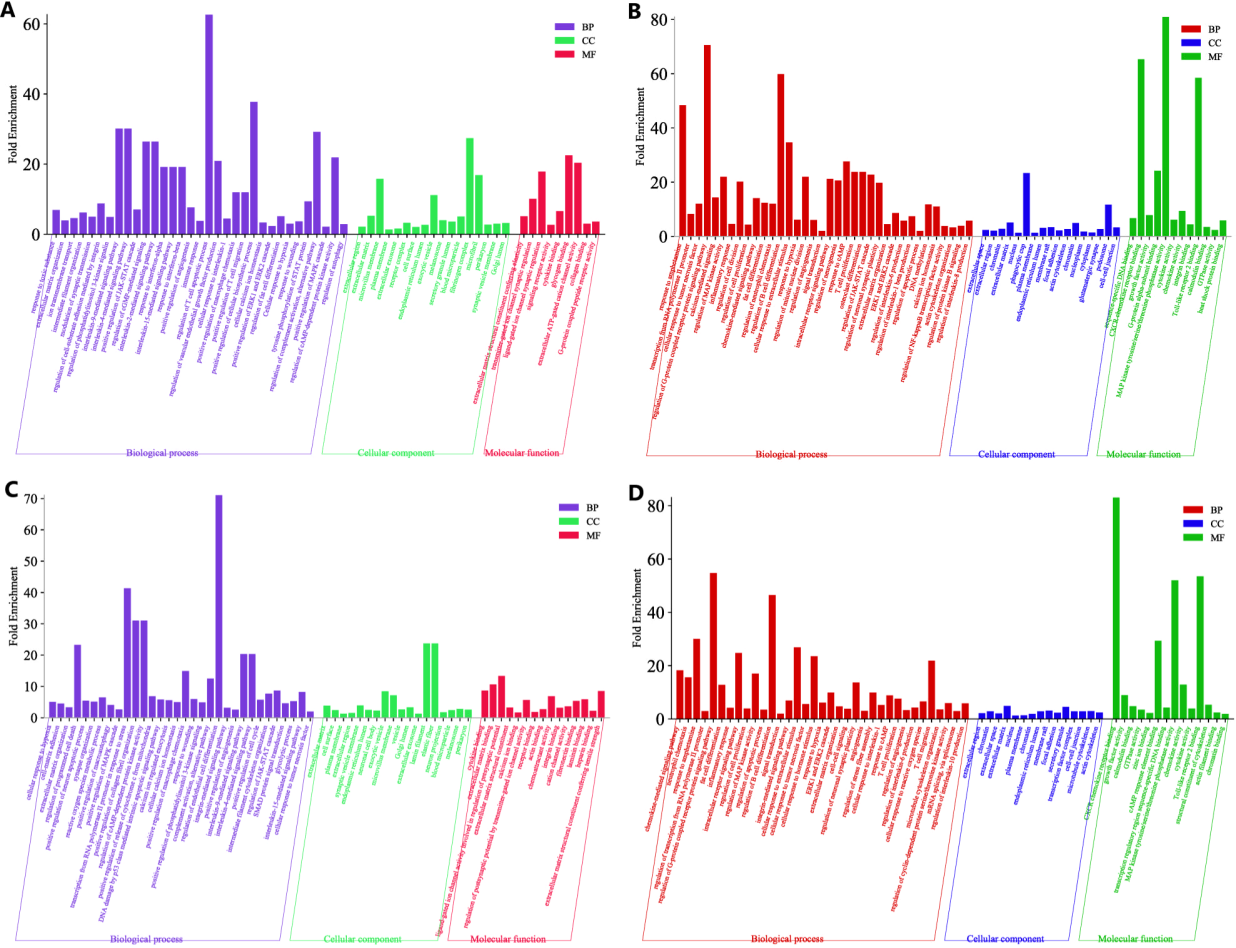
Gene Ontology representation of differentially expressed genes enriched in the host (HBMECs) during *in vitro* infection with the fungal pathogen *C neoformans/gattii* species complex, sourced from both clinical and environmental origins **(A–D)**. X-axis represents the fold enrichment of each GO terms. **(A)** Significant upregulated GO terms enriched in co-culture categories at T4 **(B)** Significant downregulated GO terms enriched in co-culture categories at T4 **(C)** Significant upregulated GO terms enriched in co-culture categories at T18 **(D)** Significant downregulated GO terms enriched in co-culture categories at T18. The data shown are representative of duplicate independent RNA-Seq experiments conducted under identical experimental conditions.

As depicted in **Figure 5**, genes associated with response to amphetamine show significant downregulation, emphasizing the host’s defense against fungal infection. Previous research has demonstrated that amphetamine disrupts BBB integrity and alters the expression of tight junction and adhesion molecules, thereby increasing susceptibility to CNS infections. This disruption of the blood brain barrier accelerates transmigration of the neurotropic fungus *Cryptococcus* spp into the brain parenchyma following systemic infection (Eugenin et al., 2013). Similarly, our study elucidates that the host responds to the infection by preserving the integrity of endothelial tight junctions, suggesting that it does not compromise BBB integrity and thus safeguards the brain from infection.

#### 3.6.1 Gene expression associated with alterations in the cellular components

We observed that the interaction between *Cryptococcus* and HBMECs within the controlled environment exhibits a complex interplay characterized by remarkable alterations in the expression profiles of genes linked to diverse cellular components. Notably, there were significant alterations in the genes associated with extracellular matrix organization, plasma membrane, Golgi, endoplasmic reticulum (ER) and nuclear component (**Table 3**). This finding aligns with the study conducted by Chen et al. which demonstrated that *C.neoformans* induces alterations in the cytoskeleton of human brain microvascular endothelial cells (Chen et al., 2003), either as the host’s effort to recognize and respond to the pathogen or the pathogen driven cellular modifications for its own advantage in the hostile environment.

**Table 3:** showing the enriched Gene Ontology terms derived from the list of differentially expressed genes associated with the alterations in the diverse cellular components within the host (HBMECs) during the early stage (4 hours) of *in vitro* infection with the fungal pathogen *C.neoformans/gattii* species complex, sourced from both clinical and environmental origins.

Cellular Component GO terms	Genes	Fold Enrichment
UPREGULATION		
extracellular matrix	SNED1, POSTN, LRRC24, ELN, BGN, TNFRSF11B, FBLN2, ADAMTS10, RELN, ADAMTS13, EMID1, FBN1, TECTA	5.25
microvillus membrane	SLC7A5, SYTL1, CA9, S100P, SLC7A11	15.78
plasma membrane	SLC48A1, IFITM1, IFITM2, GRIK5, MPL, TBC1D3E, SLC2A3, SLC7A11, MYPN, BEST1, UPK1A, OLR1, CA9, JAK3, TECTA, PAQR8, ENTPD2, UNC5B, ARRDC3, SYTL1, ISLR2, CACNA2D2, RUNDC3A, PRLR, GABRG1, SLC7A5, ALDH3A1, GPRIN2, PTP4A3, DOK7, SCNN1A, ORAI3, TRIB3, CFB, SLC47A2, PMEL, CALCRL, EPHA10, NPR1, HTRA1, SLC1A4, TNFRSF11B, SLC3A2, C3, RASD2, RELN, ERBB4, ALDH3B1, CPAMD8, REPS2, NPTX1, GRIA4, STRA6, SYT5, LIMS3, COL26A1, IFITM10, CARMIL3, TNFRSF9, HSPA6, GABRA3, SLC52A1, CP, TMEM198, SLC6A9, SYT12, FXYD2, OR2A7, MDGA1, GPR146	1.37
cell surface	LRRC24, MPL, BGN, ISLR2, SLC1A4, SLC3A2, SLC7A11, PRLR, C3, UPK1A, ADAMTS13, PSG5, BSN	2.07
endoplasmic reticulum lumen	C3, COL26A1, ADAMTS13, H6PD, STC2, IGFBP3, CP, FBN1	2.71
exocytic vesicle	SYT5, SYT12, SYTL1	11.16
melanosome	PMEL, SYTL1, SLC3A2, SLC1A4	4.01
fibrinogen complex	FGB, FGG, FN1	27.43
microfibril	EFEMP2, ADAMTS10, FBN1	16.88
perikaryon	GRIK5, SLC2A3, KLHL24, SYNPO, CRYAB, SLC8A2	2.73
Golgi lumen	MMP11, VTN, MUC1, MUC16, F10, PDGFB, BGN, PCSK6	3.19
lamin filament	NARF, LMNTD2	23.71
neuron projection	RELN, EPHA10, AVIL, GABRA3, CHAT, MPL, OPRL1, RAB39B, RGS11, MCHR1, EPHA3	1.77
DOWNREGULATION		
fat cell differentiation	NR4A2, NR4A1, EGR2, NR4A3	12.77
focal adhesion	BCAR3, EFNB2, MME, PLAUR, NEXN, LCP1, PFN1, CORO2B, TGM2	2.29
secretory granule	SRGN, PTPRN2, SCG5, CFP	4.50
transcription factor complex	NR4A2, NR4A1, JUN, NR4A3, FOS, HDAC9	2.83
cell-cell junction	COL17A1, COL13A1, LAMA1, PTPN6, STEAP1	2.86
microtubule cytoskeleton	TUBA1C, TUBA1B, PLK1, TUBB4B, HYPK, TUBA4A, TUBA8	2.98
actin cytoskeleton	CD274, SMTN, MYO5B, NEXN, SPTB, CORO2B, CORO1A, BCAR1	2.46
chromatin	NR4A2, EGR1, NR4A1, EGR2, HMGN5, NR4A3, CREB3L3, FOSB, DPF3, HES1, FOS, TGM2	2.87
membrane raft	TLR1, MME, PODXL, S1PR1, CD226, TLR6, NTSR1	3.12
phagocytic cup	TICAM2, PEAR1	23.38
nucleoplasm	EGR1, EGR2, DUSP2, NPIPA8, FOS, PWP2, PTHLH, NR4A2, NR4A1, FOSB, HES1, PTPN6, JUNB, IER2	1.79
cytoplasm	EGR1, ABHD14A-ACY1, EGR2, DUSP2, BCL2A1, DUSP1, FAXC, PTHLH, NR4A2, NR4A1, RGS2, PDZD2, HES1, PTPN6, PPIAL4G, IER2	1.45
podosome	AFAP1L1, LCP1, FERMT3	11.72

The extracellular matrix (ECM) serves as a critical interface for host-pathogen interactions influencing cellular adhesion, migration and signaling. The substantial changes observed in the ECM associated genes suggest that *Cryptococcus* may attempt to modulate cellular adhesion and thereby facilitate its adherence to HBMECs. Alterations in the genes related to the plasma membrane, which plays a central role in sensing extracellular cues may indicate either *Cryptococcus* driven modifications of the membrane structure or the host’s effort to recognize and respond to the pathogen’s presence. The changes in genes linked to ER and Golgi signifies modulations in protein processing and transportation, potentially reflecting the host’s infection induced stress or the pathogen’s manipulation of cellular trafficking for its own benefits. Furthermore, observed alterations in the nuclear gene expression points to the host’s attempt to initiate transcriptional responses to trigger immune activation or the pathogen’s manipulation to host signaling pathways to its advantage.

A noteworthy observation in our study was the significant expression levels of melanosomes in HBMECs upon encountering the pathogen *Cryptococcus*. Melanosomes known for melanin production in melanocytes was traditionally thought to be limited to melanocytes. While recent research has revealed the presence of melanosomes in other cell types such as certain immune cells and even microvascular endothelial cells (Yamaguchi and Hearing, 2014; Regazzetti et al., 2015). Melanocytes, in addition to being melanin factories, they apparently play a key role as the first line of innate immune defense against fungal infections in association with the activation of TLRs (Tapia et al., 2014). In the context of *Cryptococcus* infecting HBMECs, the positive regulation of melanosomes could potentially contribute to the host’s defense by producing melanin as a counter measure against the pathogen’s virulence mechanisms. On the other hand melanin production stands out as a key virulence factor in *Cryptococcus* species which helps to evade host immune responses by dampening the oxidative stress inflicted by immune cells and also aids in immune cell avoidance allowing it to establish infection and persist within the human host (Liu et al., 2021).

#### 3.6.2 Dual regulation of essential genes

In the process of functional enrichment analysis, another significant observation pertained to the dual regulation of specific essential genes associated with cellular components, immune responses and cell signaling pathways during *Cryptococcus*-HBMEC interaction at early and late infection time points (**Figure 5**). The differential expression of host genes during the complex interplay between *Cryptococcus* and HBMECs reflects the intricate regulatory network that *Cryptococcus* engages to adapt to the hostile environment presented by the immune system (Kronstad et al., 2012). The simultaneous upregulation and downregulation of specific genes reflect underlying gene regulatory mechanisms where *Cryptococcus* employs versatile adaptation strategies to evade host immune responses and ensure its survival within the host immune cells. However, in the context of the BBB and brain, heightened inflammation can compromise barrier integrity, facilitating further pathogen invasion and exacerbating tissue damage. This dual regulation of GO terms in challenged HBMECs highlights the complexity and context-dependent nature of host-pathogen interactions underscoring the host’s attempt to strike a balance between mounting an immune response and the avoidance of excessive inflammation and tissue damage (Cicchese et al., 2018).

### 3.7 Enrichment analysis of pathways for identified DEGs in challenged HBMECs

**Figure 6:**
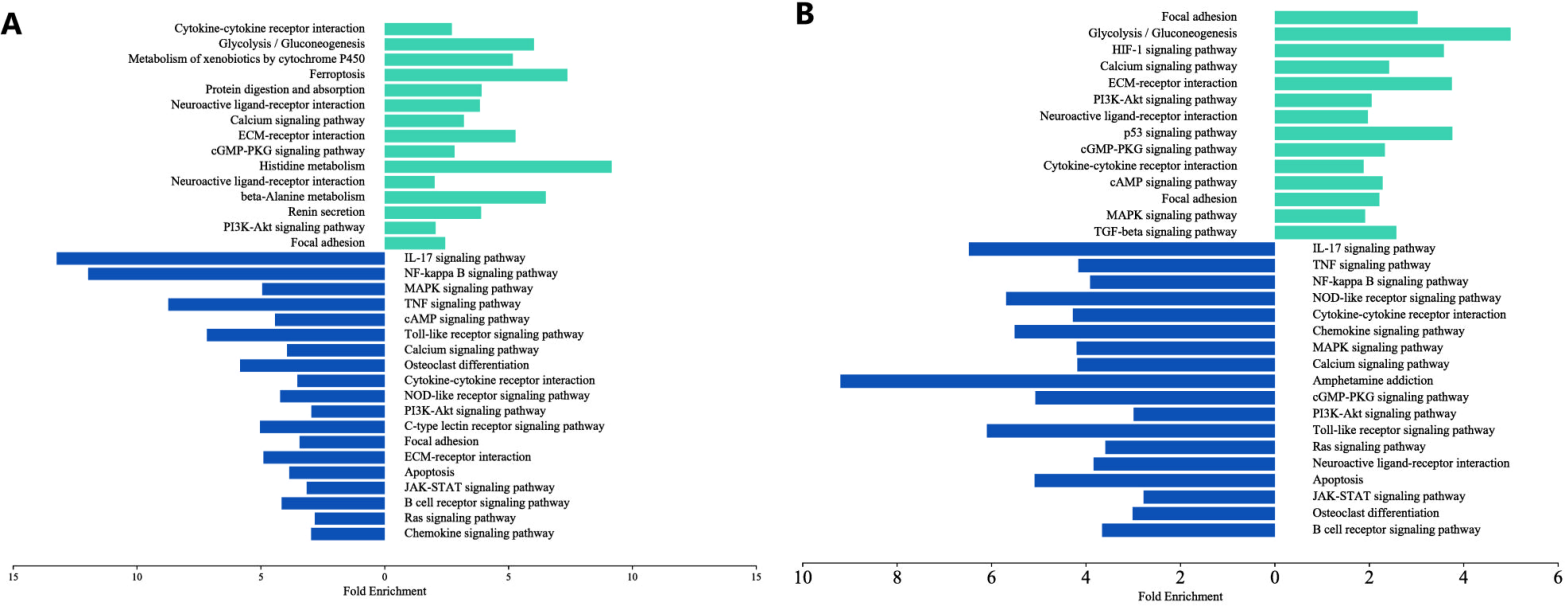
Bar plot representing the crucial function of differentially expressed essential genes obtained through pathway analysis using KEGG (Kyoto Encyclopedia of Genes and Genomes) enriched in the host (HBMECs) during interaction with the pathogen C *neoformans/gattii* species complex, isolated from both clinical and environmental sources. Few top listed significant pathways enriched from the upregulated DEGs are depicted in light blue at the top, while those enriched from the downregulated DEGs are presented in dark blue at the bottom. **(A)** Pathways enriched at early time point (T4) **(B)** Pathways enriched at later time point (T18). The data shown are representative of duplicate independent RNA-Seq experiments conducted under identical experimental conditions.

Further on to understand the molecular strategies and immune responses during infection, we analyzed KEGG pathway enrichment using DAVID, which revealed the profound association of upregulated DEGs with multiple pathways including PI3K-Akt, p53, cGMP-PKG, cAMP, MAPK, HIF-1, calcium signaling pathways. Additionally, these upregulated DEGs were found to be linked with cytokine-cytokine receptor interaction, Neuroactive ligand receptor interaction, ECM-receptor interaction and focal adhesion pathways. Conversely, the downregulated DEGs exhibited enrichment in IL-17, NF-kappa B, TNF, cAMP, JAK-STAT, MAPK, PI3K-Akt, Toll- like, NOD-like, B- cell receptor signaling pathways and osteoclast differentiation (**Figure 6**). The JAK-STAT pathway functions as the primary signaling mechanism for a multitude of cytokines including interferon, interleukins and growth factors and plays a crucial role in host defense and immune-regulation. A significant regulatory function of the phosphatidylinositol 3-kinase (PI3K) dependent extracellular signal regulated kinase 1/2 (ERK1/2) signaling pathway involves exhibiting antimicrobial activity against the yeast *Cryptococcus* (Voelz and May, 2010). In the context of integration between the major cell signaling pathways like JAK/STAT, MAPK, PI3K-dependent ERK1/2 exerts a profound impact on the immune response (Rawlings et al., 2004) and contributes in the phenomenon of combating cryptococcal infection by facilitating critical cellular processes for pathogen recognition and phagocytosis by playing a central role in orchestrating cytokine production (Yang et al., 2022).

These signaling pathways are interconnected forming a complex regulatory network that collaboratively coordinates the host’s defense mechanisms against the invading fungal pathogen. Infection with *Cryptococcus* stimulates the differentiation of Th17 cells resulting in the secretion of IL-17 which is regulated by activated CD4+T cells stimulated JAK/STAT signaling pathway there by exhibiting a profound mechanism of antifungal immunity (Guo et al., 2022). During fungal invasion, the host recognizes pathogen-associated molecular patterns (PAMPs) through Toll-like receptors (TLRs) and Nucleotide-binding oligomerization domain-like receptors (NLRs), initiating the Toll-like and NOD-like pathways, respectively, triggering a cascade of events, including the activation of NF-kappa B and MAPK pathways, leading to the production of several pro-inflammatory cytokines. Concurrently, phosphatidylinositol 3-kinase (PI3K-Akt) signaling pathway influences melanin production contributing to the pathogenesis of *Cryptococcus* spp complex (Kozubowski et al., 2009). Similarly, cAMP, cGMP-PKG, neuroactive ligand receptor signal transduction pathways promotes metabolism, cell proliferation, growth, cell survival and angiogenesis contributing to regulate the adaptive immune activation against infection.

Hypoxia-inducible factor-1 (HIF-1), triggered by hypoxia in the mammalian host, constitutes a key component of the prominent pathways engaged in innate immune responses against fungal infections (Friedrich et al., 2017). Upon encountering various cellular stress factors such as DNA damage, in response to which p53, a nuclear transcription factor gets activated in the host promoting its pro-apoptotic function. Activated p53 pathway is involved in DNA repair, senescence, cell cycle arrest and apoptosis by transactivating target genes triggering the cellular responses to limit the spread of infection (Ozaki and Nakagawara, 2011). In connection to this, in our investigation, we observed significant enrichment of the p53 pathway with a marked increase in fold change from early to later infection time points. This enrichment effectively halts damaged cell propagation, thus impeding the transmission of impaired DNA to daughter cells. Strikingly, enrichment of ferroptosis related process, a distinct form of cell death driven by lipid peroxidation and iron accumulation represents a potential regulatory mechanism associated with infectious diseases like cryptococcal meningitis suggesting a dynamic interaction between pro-apoptotic responses and cell death modalities to counteract the propagation of damaged cells and prevent the transfer of compromised genetic material (Xiao et al., 2022).

#### 3.7.1 Dual regulation of gene expression by immediate early genes

When analyzing changes in gene expression to understand how the host responds to the pathogen and how the pathogen manipulates the host's cellular processes in an *in vitro* setting, specific signaling pathways and molecular mechanisms were identified to be enriched among the down regulated differentially expressed genes as depicted in **Figure 6**. In response to a diverse array of pathophysiological or infectious stimuli, CNS can initiate the expression of immediate early genes (IEGs) which can subsequently repress their own transcription through negative feedback mechanisms (Akins et al., 1996). In our study, the prototype members of IEGs namely FOS, FOSB, FOSL1, JUN, NR4A1, EGR1, EGR2 were found to exhibit notable expression across critical signaling pathways. Notably, these IEGs function as dual-role transcription factors that are capable of both activating and repressing gene expression depending on the cellular context and the specific binding partners they interact with (Gius et al., 1990). The tug–of–war between host and the pathogen triggers a switch in the complex network of signaling pathways involving a rapid induction followed by suppression of key immune pathways as part of a regulatory feedback loop during specific stages of the infection. This dual functionality empowers IEGs to regulate a broad spectrum of biological processes in response to various stimuli.

#### 3.7.2 Anticryptococcal therapeutic targets

The functional enrichment analysis revealed the enrichment of specific pathways namely histidine metabolism, ferroptosis, IL-17 and RAS signaling pathways that displays antimicrobial activity. The anticryptococcal mechanism driven by enriched histidine metabolism in HBMECs during infection with *Cryptococcus* spp complex *in vitro* is potentiated by low pH and thereby contributes to maintaining cellular pH homeostasis creating an unfavorable environment for cryptococcal survival and proliferation (Kacprzyk et al., 2007). In addition, histidine metabolism contribute to the production of reactive oxygen species (ROS) and other free radicals, having detrimental effects on the pathogen's cellular components aimed at impeding the growth and survival of the pathogen (Helmerhorst et al., 2001). Cationic amino acids like histidine containing natural peptides such as histatins and clavanins, presents an alternative approach for the advancement of novel antifungals, commonly referred to as cationic antimicrobial peptides (CAMPs). These peptides possess a positive charge and exhibits preferential binding affinity towards negatively charged fungal membranes, typically exerting their mechanism of action through membrane penetration and the disruption of pathogenic cells (Aaghaz et al., 2023).

Ferroptosis, a form of programmed cell death characterized by its dependence on iron and lipid peroxidation, plays a pivotal role in triggering immunogenic and proinflammatory responses. This is attributed to the release of damage-associated molecular pattern molecules (DAMPs) and alarmins by ferroptotic cells, resulting in a distinct mode of regulatory cell death in infectious diseases like cryptococcal meningitis. The lipid-rich nature of *Cryptococcus* membranes makes them particularly susceptible to ferroptosis-like mechanisms that disrupt lipid bilayers, causing cell rupture. Ferroptosis is intricately dependent on reactive oxygen species and iron, with disruption in intracellular iron metabolism or glutathione peroxidation pathways resulting in lipid reactive oxygen species accumulation and subsequent cell death. The potential involvement of ferroptosis in the pathogenesis of infectious diseases, such as cryptococcal meningitis, offers a novel avenue for therapeutic intervention, suggesting the exploration of more potent adjuvant treatments against cryptococcal meningitis (Xu et al., 2021).

Previous research has demonstrated that the IL-17 pathway governs antifungal immunity by inducing the upregulation of pro-inflammatory cytokines like IL-6, neutrophil-recruiting chemokines such as CXCL1 and CXCL5, and antimicrobial peptides like defensins, collectively working to constrain fungal overgrowth (Conti and Gaffen, 2015). Our study aligns with these findings, as we observed the enrichment of the IL-17 signaling pathway in HBMECs during *Cryptococcus* infection, associated with the regulation of proinflammatory cytokines including CXCL6, IL6, CXCL8, CXCL1, CXCL3, CXCL2, CCL20, and IL1B. This enrichment holds promise for therapeutic strategies against infectious diseases like cryptococcal meningitis. Through an *in vitro* mechanistic study, it was shown that activated CD4+ T cells were the primary source of IL-17 production, and Th17 cell differentiation was modulated by diverse signaling pathways, with the JAK2/STAT3 pathway playing a pivotal role. This study also underscores the significance of CD4+ T cells in antifungal immunity, suggests IL-17 as a diagnostic biomarker for cryptococcal infection, and proposes STAT3 as a potential target for antifungal therapies (Guo et al., 2022).

Another noteworthy observation in our study is the enrichment of Ras protein signaling pathway which is commonly associated with cellular growth, proliferation and survival. However, the activation of this pathway can indirectly contribute to antifungal defense mechanisms within the host in response to fungal infections including those caused by *Cryptococcus* species. Considering its involvement in the virulence and pathogenic characteristics of various yeast and fungal species, Ras protein signaling pathway upholds promise as a potential antifungal drug target (Fortwendel, 2012). Additionally, studies have demonstrated that modulating Ras signaling serves as a critical regulatory node for triggering apoptosis in the budding yeast *S. cerevisiae* (Gourlay and Ayscough, 2005; Gourlay and Ayscough, 2006; Leadsham et al., 2009) and *C. albicans* (Phillips et al., 2006). These findings further imply that pharmacological alterations of Ras protein signaling could be of considerable advantage to induce yeast cell death. While some current antifungals indirectly affect fungal Ras signaling, like amphotericin B that enters fungal membranes containing ergosterol, creating aqueous pores, causing localized thinning of the bilayer and thereby compels lipid-anchored Ras proteins into sterol-rich lipid rafts, facilitating their interactions with downstream proteins and promoting the initiation of signaling pathways. Pharmacologically manipulated enhanced Ras protein signaling triggers yeast apoptosis and oxidative damage by the production of reactive oxygen species via the cAMP-PKA pathway (Pentland et al., 2018).

### 3.8 Fungal transcriptomic responses and virulence-associated pathways

We investigated the early and late differential gene expression changes exhibited by the fungal pathogen *C.neoformans/ gattii* spp complex isolated from the clinical and environmental sources during *in vitro* infection of HBMECs. The pathogen appears to adopt strategic mechanisms to enhance resistance against host immune defenses, which are key determinants of its virulence. To understand the gene expression responses elicited by the pathogen during host interaction we compared pathogen-derived reads from all eight treated co-culture categories described above (**Table 2**) against their corresponding isolates grown in laboratory culture medium.

**Figure 7:**
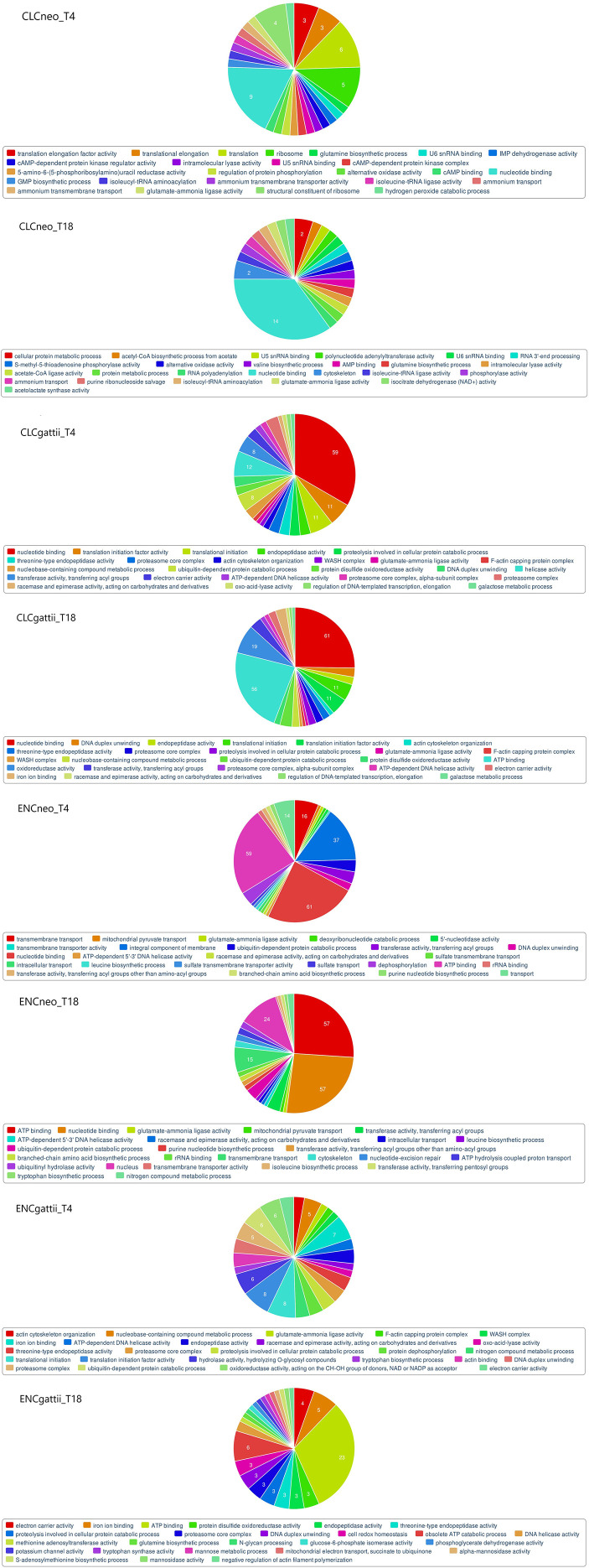
Functional categorization of differentially expressed genes derived from the fungal pathogen *Cryptococcus neoformans/ gattii* species complex originating from both clinical and environmental sources. The categorization was conducted across eight described treated co-culture conditions by Gene Ontology database using FungiFun2 tool, showcasing the 25 most significantly enriched categories within each group. The selection criterion to sort the DEGs considered the cut off of ≤ 0.05 for p-values, ≤ 0.05 for FDR and an absolute log2FoldChange value ≥ 1.5 for up regulated and ≤ -1.5 for down regulated DEGs. The data shown are representative of duplicate independent RNA-Seq experiments conducted under identical experimental conditions.

In *Cryptococcus*, mapped reads ranged between 0.07% – 0.15% when aligned to the custom built reference genome from fungi ensemble. This low mapping rate aligns with the findings from previous dual RNA-Seq studies and supports the consistency of our results (Petrucelli et al., 2018; Hayward et al., 2021; López-Agudelo et al., 2022). This comparative analysis revealed a range of genes found to be differentially expressed in the pathogen across treated co-culture groups as shown in **Table 4**. We characterized the significant up and down regulated genes by Gene Ontology functional classification database using FungiFun2 and found 25 most significant enriched categories among all eight treated groups involved in diverse biological processes, metabolism and cellular remodeling (**Figure 7**).

**Table 4:** Considerable range of differentially expressed genes in the pathogen across various treated co-culture categories in comparison with the laboratory media control.

Treated conditions	Up regulated gene	Down regulated gene
CLCneo_T4	34	25
CLCneo_T18	64	39
CLCgattii_T4	03	545
CLCgattii_T18	03	573
ENCneo_T4	04	706
ENCneo_T18	05	593
ENCgattii_T4	04	444
ENCgattii_T18	21	129

The analysis employed cut off of ≤ 0.05 for p-values, ≤ 0.05 for false discovery rate (FDR) and an absolute log2FoldChange value ≥ 1.5 for up regulated and ≤ -1.5 for down regulated significant profiling of the genes that are differentially expressed.

The Gene Ontology functional enrichment analysis revealed critical biological processes and pathways contributing to the virulence and pathogenicity of *Cryptococcus* species complex. One of the significant findings was the enrichment of genes related to cytoskeleton organization and autophagy. The importance of the WASH complex, necessary for autophagosome formation, was highlighted. Autophagy, a degradative pathway, plays a crucial role in recycling cellular components and providing amino acids essential for survival, particularly under stress conditions (King et al., 2013; Jiang et al., 2020).

The study emphasized the role of genes involved in cell wall remodeling, carbohydrate and nitrogen metabolism, Ubiquitin mediated proteolysis, metabolite and energy generation, and mitochondrial organization. These processes are essential for establishing and progressing infections, as well as modulating the production and activity of virulence factors, such as fungal cell wall structure and melanin (Ries et al., 2018; Cao and Xue, 2021). Ribosomal modulation and proteasome activity were found to be vital for the degradation of cellular proteins and the synthesis of the polysaccharide capsule in *Cryptococcus* during the early stages of infection (Freitas et al., 2022).

Ubiquitin, a conserved protein, plays a central role in various fundamental cellular processes, contributing to the growth and pathogenicity of *Cryptococcus* species (Liu and Xue, 2011). Inositol catabolism was identified as having a role in capsule growth and contributing to the pathogenesis of CNS cryptococcosis. The presence of abundant inositol in human and animal brains, coupled with the advanced inositol acquisition system in the yeast *Cryptococcus*, indicates a potential link between host inositol utilization and the development of cryptococcal meningitis. A study demonstrates the significant role of inositol in facilitating *Cryptococcus* traversal across the blood-brain barrier in both *in vitro* human BBB models and *in vivo* animal models (Liu et al., 2013).

Gene expression related to cellular components, including actin cytoskeleton organization, F-actin capping protein complex, mitochondrial and nuclear remodeling, exhibited significant modulation during infection. These alterations likely influence the structural and functional adaptations of *Cryptococcus* within the host environment. The study also revealed the regulation of genes associated with stress responses, energy metabolism, and protein modification, indicating the pathogen's ability to adapt to the challenges posed by the host environment. Notably, genes involved in translation, DNA-templated transcription, and elongation were differentially regulated, suggesting potential adaptive mechanisms employed by *Cryptococcus* species during infection.

### 3.9 Substantial genomic variation at 4 h and 18 h post-infection

In our study, conducted with an aim at elucidating the intricate dynamics underlying host-pathogen interactions, we examined the expression profiles of the top-listed genes, differentially expressed at the 4^th^ hour time point, exhibited consistent expression levels at the 18^th^ hour time point in the infecting pathogen *Cryptococcus* species complex. Our findings revealed that key signaling pathways, known for their crucial roles in the immunopathogenesis of cryptococcal meningitis, exhibited a remarkable degree of consistency in their expression patterns at both the 4^th^ and 18^th^ hour time points within HBMECs, an *in vitro* cell culture system. This finding emphasizes that immune signaling pathways enriched in HBMECs i.e. the host cells under investigation, as well as virulence-related attributes that are differentially regulated in the pathogen, play pivotal roles throughout the early and later time points of infection process. These results underscore the significant contributions of these pathways and the associated genes in shaping the complex interplay between the host and the pathogen over the course of infection.

### 3.10 Comparative expression profiles of clinical vs. Environmental isolates

After investigating the virulence-related attributes exhibited by the pathogen and the immune responses displayed by the host during the critical events of infection under controlled *in vitro* experimental conditions, we proceeded to assess the relative competency of environmental isolates of cryptococci in infecting HBMECs. HBMECs constitute a significant component of the human blood-brain barrier and are integral to the establishment of central nervous system infections. We assessed the invasion capability and virulence potential of environmental strains of cryptococci in comparison to that of clinical isolates and concurrently examined alterations in the regulation of gene expression associated with virulence factors when infecting HBMECs *in vitro*.

Our findings demonstrated that the naive environmental isolates of cryptococci despite lacking prior exposure to the human host milieu, exhibited a comparable infectivity and virulence profile to that of clinical isolates when infecting HBMECs. This was supported by the observed expression of various virulence-related elements associated with melanization, polysaccharide capsule formation, metabolite and energy generation, apoptotic and autophagy related functions in clinical as well as environmental isolates of cryptococci which are essential for establishing and progressing infections. Furthermore, the gene expression related to the alterations in the cellular components, potentially influencing the structural and functional adaptive mechanisms employed by cryptococci within the host environment exhibited remarkable similarity between clinical and environmental isolates. Notably, both clinical and environmental isolates of *Cryptococcus* demonstrated comparable impacts on the host by inducing the activation of essential immune signalling pathways within the host during *in vitro* infection.

### 3.11 DEGs validation

**Figure 8:**
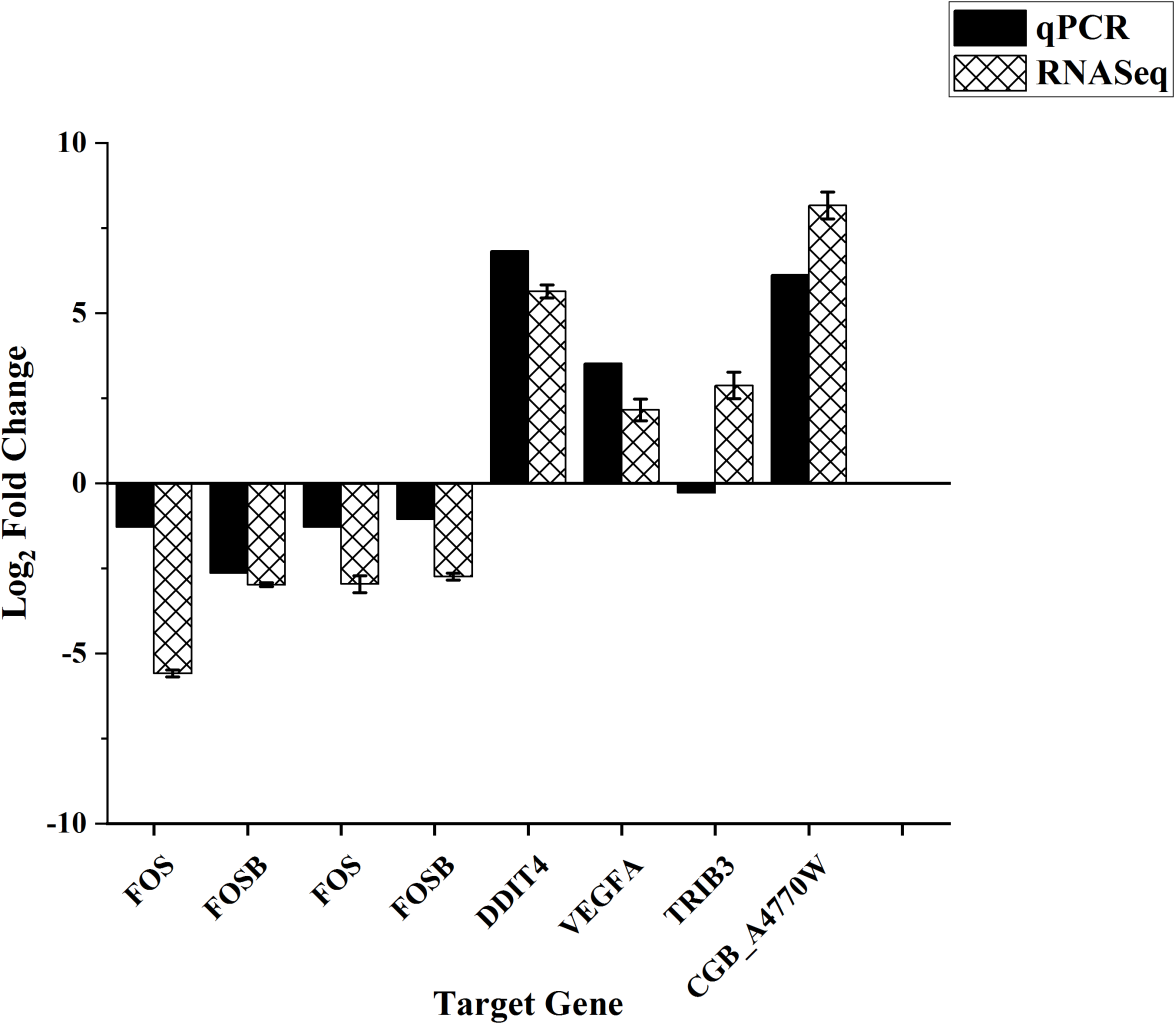
Bar plot depicting the correlation between Dual RNASeq and qPCR. The gene expression results from RNA-seq exhibited a robust correlation (r = 0.8858, p-value = 0.00341) with the gene expression values obtained through qPCR.

To evaluate the correlation between dual RNA-seq and qPCR, nine genes were selected for validation—seven from the host (HBMECs) and two from the pathogen (*Cryptococcus*). **Figure 8** illustrates the comparison of Log2 fold change values obtained through both techniques. The correlation between the two methods was assessed using Pearson’s correlation test. The gene expression results from RNA-seq exhibited a robust correlation (r = 0.8858, p-value = 0.00341) with the gene expression values obtained through qPCR. One pathogen target gene (CGB_A4480W) was excluded from the correlation analysis due to significant variation in Log2Foldchange values (12.66 and 3.09) obtained by RNA-seq and qPCR. No correlation was observed between RNA-seq and qPCR for the host target gene TRIB3, indicating a potential technical error in the experiment. However, upon comparing the remaining seven targets out of the overall nine, our findings suggest that sequencing yielded dependable outcomes, underscoring the accuracy and reproducibility of the techniques.

## 4 Discussion

This study represents a comprehensive preliminary investigation into the early events of *Cryptococcus*–host interaction, integrating ultrastructural and transcriptomic data to provide mechanistic insights into endothelial responses during fungal invasion. We demonstrated that both clinical and environmental isolates of the *Cryptococcus neoformans/gattii* species complex can invade HBMECs, inducing significant ultrastructural changes at both 4 and 18 hours post-infection. These alterations involved various subcellular organelles, reflecting the host's defense mechanisms and the pathogen's strategies for survival and propagation. One such response involves the formation of membrane rufflings, which are temporary protrusions of the cell membrane resulting from alterations in the actin cytoskeleton dynamics. It is an integral part of the host cell's response to the presence of the pathogen that facilitates pathogen adherence and internalization (Filler and Sheppard, 2006). In parallel, infected HBMECs exhibited extensive formation of microvilli which are finger-like projections on the surface area of endothelium that may enhance the cell's capacity to interact with the external environment, potentially augment pathogen detection and capture of cryptococcal cells (Eugène et al., 2002).

The formation of autophagosome within the HBMECs is a strategic move in the host’s battle against the pathogen. These specialized vesicles encapsulate potentially harmful damaged organelles and intracellular pathogens, thereby marking them for subsequent degradation functioning to eliminate the pathogen (Riebisch et al., 2021). Autophagy, a highly regulated process can modulate the host's immune response by influencing the presentation of pathogen-derived antigens, contributing to the activation of immune cells and the production of immune-signaling molecules like cytokines and chemokines (Xu and Eissa, 2010; Jiang et al., 2019). However, *Cryptococcus* has evolved sophisticated strategies to resist or evade autophagic capture and degradation, highlighting the ever-evolving arms race between host and pathogen in the realm of infection.

Mitochondria play essential roles in innate immune responses triggering the expression of pro-inflammatory cytokines through the NF-kB pathway and contribute to the generation of ROS as part of the cellular defense against pathogenic infections (Fu et al., 2022). The observed alterations could imply on impairment in the regulation of inflammatory response against the invading pathogen. Conversely, the observed mitochondrial alterations may represent a counter measure by the host cell in response to the infection, potentially aimed at limiting the replication and spread of *Cryptococcus* or initiating mitophagy (Khan et al., 2020) or apoptotic pathways as a defense mechanism.

Alterations in Golgi morphology may potentially disrupt the Golgi's ability to sort and modify proteins that could consequently impact the secretion of essential molecules involved in cellular signaling and immune responses and obstruct the post-translational modification in the host cell (Liu et al., 2021). Dysfunction of the Golgi apparatus has been documented in various infections caused by pathogens such as Orf virus (Rohde et al., 2012), Chlamydia trachomatis (Heuer et al., 2009; Pruneda et al., 2018), Hepatitis C virus (Hansen et al., 2017), Human Rhinovirus (Mousnier et al., 2014), and Rickettsia rickettsii (Aistleitner et al., 2020).

Morphological changes in the ER, suggest that cryptococcal infection can induce ER stress in HBMECs. When ER becomes stressed, it typically responds by dilating or expanding to cope with the increased accumulation of misfolded or misassembled proteins within the ER lumen. This ER dilation may be an indicator of cellular attempts to manage the stress induced by the infection possibly initiating the unfolded protein response (UPR), a cellular signaling pathway aimed at restoring ER homeostasis (Frabutt and Zheng, 2016; Glingston et al., 2019). The observed changes could be a manifestation of the UPR leading to immunomodulatory effects that influence the production of cytokines and other immune-related factors, potentially affecting the host's immune response to the infection (Smith, 2018).

The observed nuclear morphological changes in HBMECs likely signify stress responses triggered by cryptococcal infection. Such alterations can influence gene expression thereby affecting the regulation of specific genes associated with immune responses, inflammation and various other cellular processes in HBMECs. Disruption of the nuclear envelope, which is vital for nucleocytoplasmic transport suggests impaired cellular regulation under pathogenic stress. These changes may also impact immune signaling that could influence the activity of transcription factors and nuclear proteins affecting cytokine and chemokine production during infection (Nastasi et al., 2020; Selezneva et al., 2022). Cytoplasmic vacuolation could be a cellular response to infection and may serve as a containment strategy, aimed at sequestering, neutralizing, and ultimately degrading the invading fungal cells. However, it’s important to note that *Cryptococcus* may also employ its own strategies to evade or circumvent these host defenses in its quest for persistence. The stress fibers assist in facilitating the movement of pathogen containing vesicles within the cell, potentially directing them toward lysosomes for degradation aiding in efficient engulfment of the pathogen (Franco and Shuman, 2012).

Dual RNA-Seq profiling revealed a dynamic transcriptional interplay between *Cryptococcus* spp. and HBMECs during *in vitro* infection. Host cells exhibited differential regulation of key immune pathways including PI3K-Akt, JAK-STAT, MAPK, NF-κB, and IL-17 signaling, reflecting a tightly controlled inflammatory response to maintain BBB integrity while combating the fungal invasion. Notably, immediate early genes such as *FOS*, *JUN*, and *EGR1* were prominently expressed, suggesting rapid transcriptional activation. Simultaneously, genes linked to autophagy, ferroptosis, and cytokine signaling were modulated, indicating a multilayered defense strategy. On the pathogen side, clinical and environmental isolates showed similar expression of virulence-related genes, including those involved in melanin production, capsule biosynthesis, and stress response. These findings highlight a conserved host response to cryptococcal infection and reveal that environmental strains possess comparable pathogenic potential. The study underscores the significance of early immune signaling events at the BBB and provides a foundation for identifying molecular targets for antifungal intervention.

Further reinforcing our transcriptomic findings, ultrastructural observations obtained via TEM revealed hallmark cellular alterations in infected HBMECs. Notably, the subcellular changes observed through TEM correspond with the differential expression of host genes associated with cellular components, as identified through RNA-Seq. This concordance between structural and transcriptomic evidence provides mutual validation, strengthening the reliability of our findings and underscoring the biological relevance of the host-pathogen interactions characterized in this study. These findings suggest that endothelial cells of the BBB deploy comparable defense mechanisms against *Cryptococcus*, irrespective of whether the invading isolate is of clinical or environmental origin. Significantly, both isolate types demonstrated equivalent invasive capacity and induced comparable morphological adaptations in HBMECs, underscoring their shared pathogenic potential. Taken together, this integrative study provides novel insight into the early host-pathogen dynamics at the BBB. The dual application of TEM and dual RNA-Seq in tandem provides a robust framework for dissecting host-fungal interactions and lays the groundwork for future functional and translational studies targeting improved therapeutic strategies for cryptococcal meningitis.

However, it is essential to acknowledge that *in vitro* cell culture experiments have inherent limitations compared to *in vivo* conditions, as they do not fully replicate the complexity of host-pathogen interactions within a living organism when studying complex biological processes like the BBB. Additionally, functional validation of identified differentially expressed genes and pathways was beyond the current scope of the study. Enhanced recovery of pathogen-derived reads aligning to the custom-built cryptococcal reference genome within the co-culture libraries could have substantially improved the depth and accuracy of the pathogen's differential gene expression analysis.

Future studies can address these limitations by adopting an *in vitro* 3D model of the blood-brain barrier and optimizing the sequencing of pathogen-derived reads. These advancements would complement our current findings and provide supplementary insights. In addition studies on microRNA can significantly contribute to our findings by unraveling additional layers of the host-pathogen interaction dynamics. Examining microRNA expression profiles in response to *Cryptococcus* invasion could elucidate the regulatory mechanisms governing the host's immune responses and help identify novel therapeutic targets or diagnostic biomarkers for cryptococcal meningitis.

## Data Availability

The datasets presented in this study can be found in online repositories. The names of the repository/repositories and accession number(s) can be found below: https://www.ncbi.nlm.nih.gov/, PRJNA1068840.
